# Effect of inspired oxygen fraction during anesthesia on inflammation and antioxidant enzyme activity in the mouse cortex and hippocampus

**DOI:** 10.3389/fnagi.2026.1761281

**Published:** 2026-03-04

**Authors:** Jee-Eun Chang, Elliot H. Lee, Soo-Jin Oh, Jin-Young Hwang

**Affiliations:** 1 Department of Anesthesiology and Pain Medicine, SMG-SNU Boramae Medical Center, College of Medicine, Seoul National University, Seoul, Republic of Korea; 2 Brain Science Institute, Korea Institute of Science and Technology, Seoul, Republic of Korea

**Keywords:** anesthesia, antioxidant enzyme activity, cortex, hippocampus, inflammation, inspired oxygen

## Abstract

**Introduction:**

Although high inspired oxygen fraction (FiO₂) is used during anesthesia to prevent hypoxemia, the effect of different oxygen fraction on the brain remains unclear. This study aims to evaluate whether different inspired oxygen fractions (FiO₂ 30% vs. 80%) during anesthesia affect inflammation and antioxidant enzyme activity in the cortex and hippocampus of young and aged mice.

**Methods:**

Young and old mice were anesthetized with sevoflurane at FiO₂ 30% or 80% for 3 h. Mice in the control group were exposed to medical air (FiO₂ 21%) for 3 h. Cytokine and superoxide dismutase (SOD) assays were performed on the cortex and hippocampus samples after anesthesia.

**Results:**

The IL-1β level in the hippocampus was significantly higher in the FiO₂ 80% group compared with controls [5.0 (4.0–6.9) pg. mL^−1^ vs. 2.3 (1.6–2.7) pg. mL^−1^; adjusted *p* = 0.032], whereas no significant differences were observed in IL-1β levels between the control and FiO₂ 30% groups [adjusted *p* = 0.164] or the FiO₂ 30% and FiO₂ 80% groups [adjusted *p* = 0.390]. Except for IL-1β in the hippocampus, no significant differences in the cytokine levels and SOD activities were observed among the groups according to the inspired oxygen fraction in either brain region or age group [*p* > 0.05].

**Discussion:**

Only 80% oxygen increased hippocampal IL-1β compared with controls, suggesting region-specific vulnerability to oxygen-induced neuroinflammation. However, no significant differences between FiO₂ levels (30% vs. 80%) indicate a limited neuroinflammatory impact under 3 h of anesthesia. Further studies with longer exposure and surgical conditions are needed to clarify the clinical implications.

## Introduction

Although a high inspired oxygen fraction (FiO₂) is commonly used during general anesthesia to prevent hypoxemia, the optimal FiO₂ during anesthesia remains unclear. In surgical settings, an 80% FiO₂ significantly reduces the surgical site infection compared with a standard FiO₂ of 30% or 35% (Qadan et al., 2010; Allegranzi et al., 2016). However, supraphysiologic oxygen levels may have unintended neurophysiological consequences. Hyperoxia may increase the production of reactive oxygen species (ROS), leading to oxidative stress and cellular injury in various tissues, including the brain (Singer et al., 2021). Excessive oxygen administration may also influence inflammatory responses (Helmerhorst et al., 2015). A preclinical study showed that hyperoxia alone provoked inflammatory responses under mechanical ventilation, even in the absence of surgical injury (Helmerhorst et al., 2017). Currently, the direct effects of high FiO₂ administered during anesthesia, independent of surgical intervention, remain incompletely understood.

The hippocampus and cerebral cortex are key brain regions involved in memory formation, learning, and cognitive processing, and are known to be particularly vulnerable to oxidative and inflammatory insults when the oxygen concentration is altered (Micaux et al., 2024; Cervos-Navarro and Diemer, 1991). An experimental study examining the effect of mild hyperoxia (FiO₂ 30%) and excessive hyperoxia (FiO₂ 100%) on rat hippocampal neurons suggested that short-term mild and excessive hyperoxia induced a detrimental effect on the hippocampal neurons (Hencz et al., 2024). Several experimental studies also showed that supraphysiological oxygen administration results in neuronal damage in the brain of developing animals (Yis et al., 2008; Felderhoff-Mueser et al., 2004; Lithopoulos et al., 2022; Gerstner et al., 2008).

Oxidative stress increases with age (Ionescu-Tucker and Cotman, 2021), and the effects of hyperoxia on inflammation and oxidative stress in the hippocampus and cerebral cortex may differ with age. However, these effects under anesthetic conditions remains unclear. This study aims to evaluate how varying inspired oxygen fractions (FiO₂ 30% vs. 80%) during anesthesia affect the inflammation and antioxidant activity in the cortex and hippocampus of young and old mice.

## Methods

### Animal care and preparation

All animal experiments and animal care were conducted in accordance with the Guide for the Care and Use of Laboratory Animals. The experimental protocol was approved by the Institutional Animal Care and Use Committee of the SMG-SNU Boramae Medical Center (no. 2025–0030). Four to five mice of the same group were raised in a cage with 12-h cycles of light and dark and had free access to food and water. The temperature of the nurturing room was maintained at 23 °C with relative humidity 40 to 60%.

### Experimental groups

**Figure 1:**
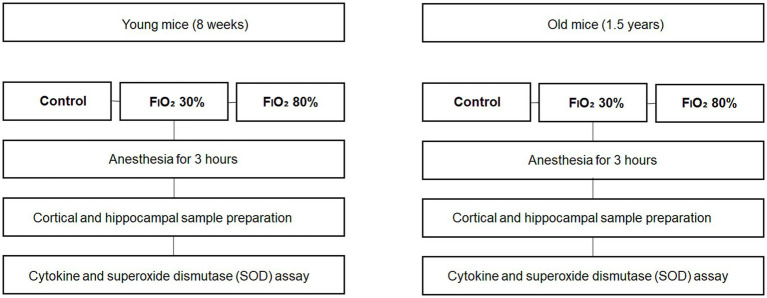
A schematic figure outlining the experimental workflow.

Male mice (C57BL/6) aged eight weeks (young group) or 1.5 years (old group) were used. The mice were assigned to one of the following six groups (*n* = 9 each): young-control, young-30%, young-80%, old-control, old-30%, or old-80%. During anesthesia, oxygen was administrated at an FiO₂ of 30% (30% group), FiO₂ of 80% (80% group), or FiO₂ of 21% (medical air, control group) (Figure 1).

### Experiments

The mice were individually placed in an air-tight transparent acrylic chambers. All mice were anesthetized with sevoflurane 1 MAC at an FiO₂ of 30% or 80% for 3 h, and mice in the control group were placed in an acrylic chamber with medical air (FiO₂ of 21%) for 3 h. Anesthetics and oxygen were supplied at a flow rate of 3.0 L min^−1^ through the inlet of the chamber. The gas was eliminated into a suction scavenger at a constant rate through the outlet. The anesthetic concentration of the anesthetics was continuously monitored using a gas analyzer (Datex-Ohmeda S/5 Anaesthesia Monitor, GE Healthcare, Chicago, IL, USA). The chamber was warmed at 37 °C with a heating pad.

### Sample preparation

Decapitation was performed immediately after 3 h of anesthesia. The brain was promptly excised from the skull, divided into ipsilateral and contralateral hemispheres, and samples of the cortex and hippocampus were obtained. The cortex and hippocampal samples were separately homogenized in lysis buffer (tissue weight/volume ratio 1:20), consisting of 0.1 M NaCl, 0.01 M Tris/HCL, 1% Triton X at pH adjusted 7.4, and centrifuged to remove debris for 5 min at 4 °C. The supernatant was collected, aliquoted, and stored at −80 °C. Sample collection was consistently performed in the early afternoon (between 12:00 and 13:00) across all experimental groups to minimize the potential impact of diurnal variation.

### Cytokine and superoxide dismutase (SOD) assay

Cytokines for brain samples in each group [IFN-*γ* (interferon-γ), IL-1*α* (interleukin-1α), IL-1β, IL-2, IL-4, IL-5, IL-6, IL-9, IL-12, IL-13, IL-17, and TNF-α (tumor necrosis factor-α)] were evaluated using MILLIPLEX® Mouse Cytokine/Chemokine Magnetic Bead Panel kit (Millipore, Billerica, MA, USA) according to the manufacturer’s instructions. Briefly, the standards, quality controls, and diluted samples were incubated with fluorescent-coded magnetic beads conjugated to analyte-specific antibodies. Following incubation overnight at 4 °C, unbound materials were removed through a series of washing steps. Biotinylated detection antibodies and streptavidin−phycoerythrin were added sequentially, followed by additional washes. The bead-associated fluorescence was measured using a Luminex® instrument, and analyte concentrations were calculated by fitting standard curves using a five-parameter logistic regression model.

The superoxide dismutase (SOD) assay is a biochemical technique used to quantify the activity of SOD, an essential antioxidant enzyme that catalyzes the conversion of superoxide radicals to molecular oxygen and hydrogen peroxide, to assess the cellular response to oxidative stress. The SOD activity was measured using colorimetric Superoxide Dismutase Activity Assay Kit (Cayman, Ann Arbor, MI, USA) according to the manufacturer’s instructions. This assay is based on the competition between SOD and a tetrazolium salt for superoxide radicals generated by the xanthine/xanthine oxidase system. Briefly, the standards and diluted samples were added to a 96-well plate along with a radical detector. The reaction was initiated by adding xanthine oxidase and incubating for 30 min at room temperature with gentle shaking. The absorbance was measured at 450 nm using a microplate reader. SOD activity, was calculated by comparing the sample absorbance values with a standard curve, with lower absorbance indicating higher SOD activity.

### Statistical analysis

Statistical analyses were performed using SPSS version 29 (IBM Corp., Armonk, NY, USA) and R software version 4.3.2 (R Foundation for Statistical Computing, Vienna, Austria). Normality was assessed using the Shapiro–Wilk test. Data are presented as the median [interquartile range]. Differences in cytokine levels and SOD activity among the control, FiO₂ 30%, and FiO₂ 80% groups were evaluated using the Kruskal–Wallis test, followed by Dunn’s *post hoc* test with Holm correction. Differences in cytokine levels and SOD activity between young and old mice at each inspired oxygen concentration were assessed using the Mann–Whitney U test. A *p-*value < 0.05 was considered statistically significant.

## Results

**Figure 2:**
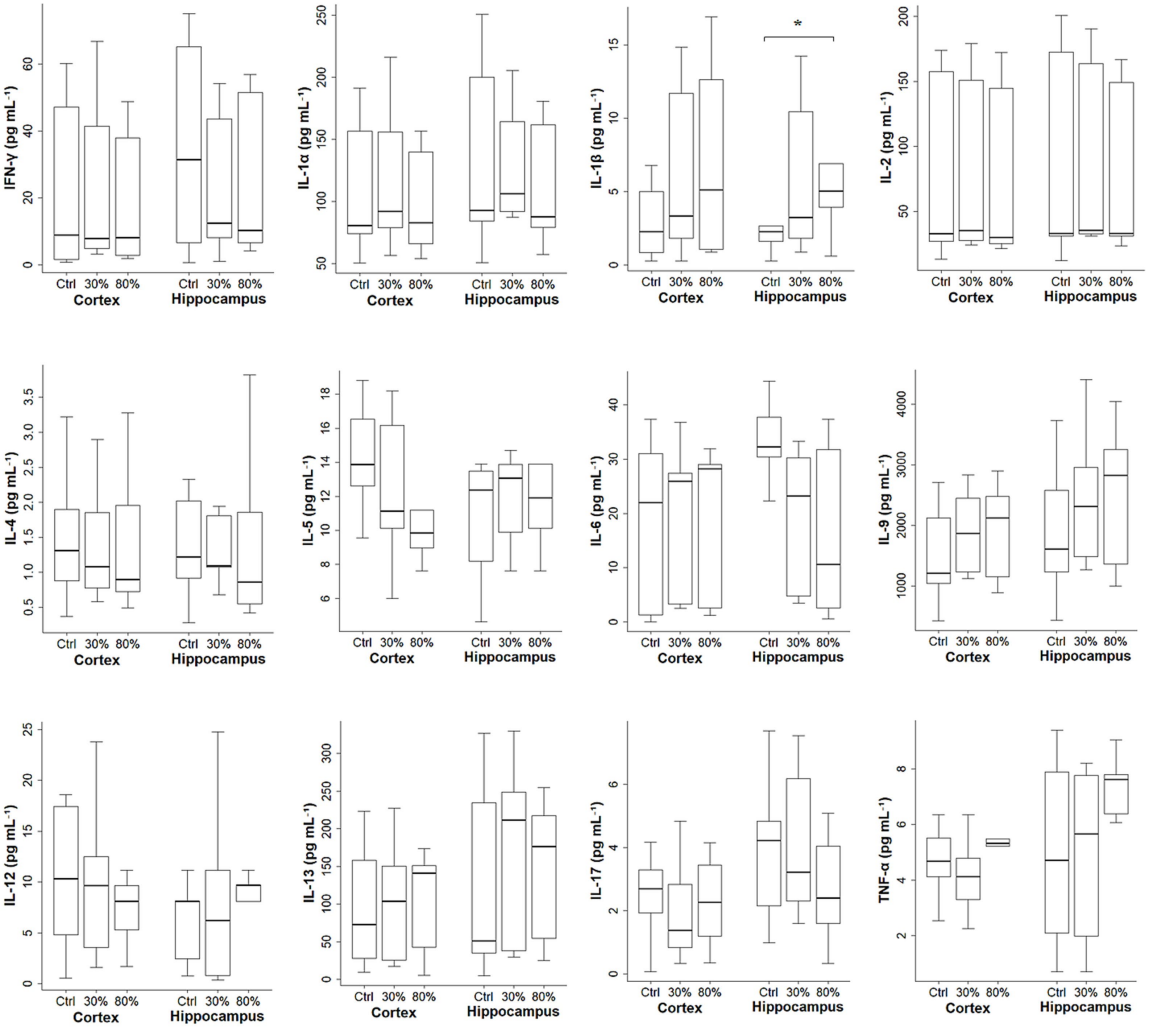
Cytokine levels based on oxygen fraction in all age groups (regardless of age). The data are presented as box plots. The horizontal box lines represent the 75th, 50th, and 25th percentiles. The stems represent the 90th and 10th percentiles. IFN-*γ*, interferon-γ; IL, interleukin; TNF-*α*, tumor necrosis factor-α. *p = 0.034 among the control vs. FiO₂ 30% vs. FiO₂ 80%; *post hoc* comparisons (control vs. 80%, adjusted *p* = 0.032; control vs. FiO₂ 30%, adjusted *p* = 0.164; FiO₂ 30% vs. FiO₂ 80%, adjusted *p* = 0.390).

**Figure 3:**
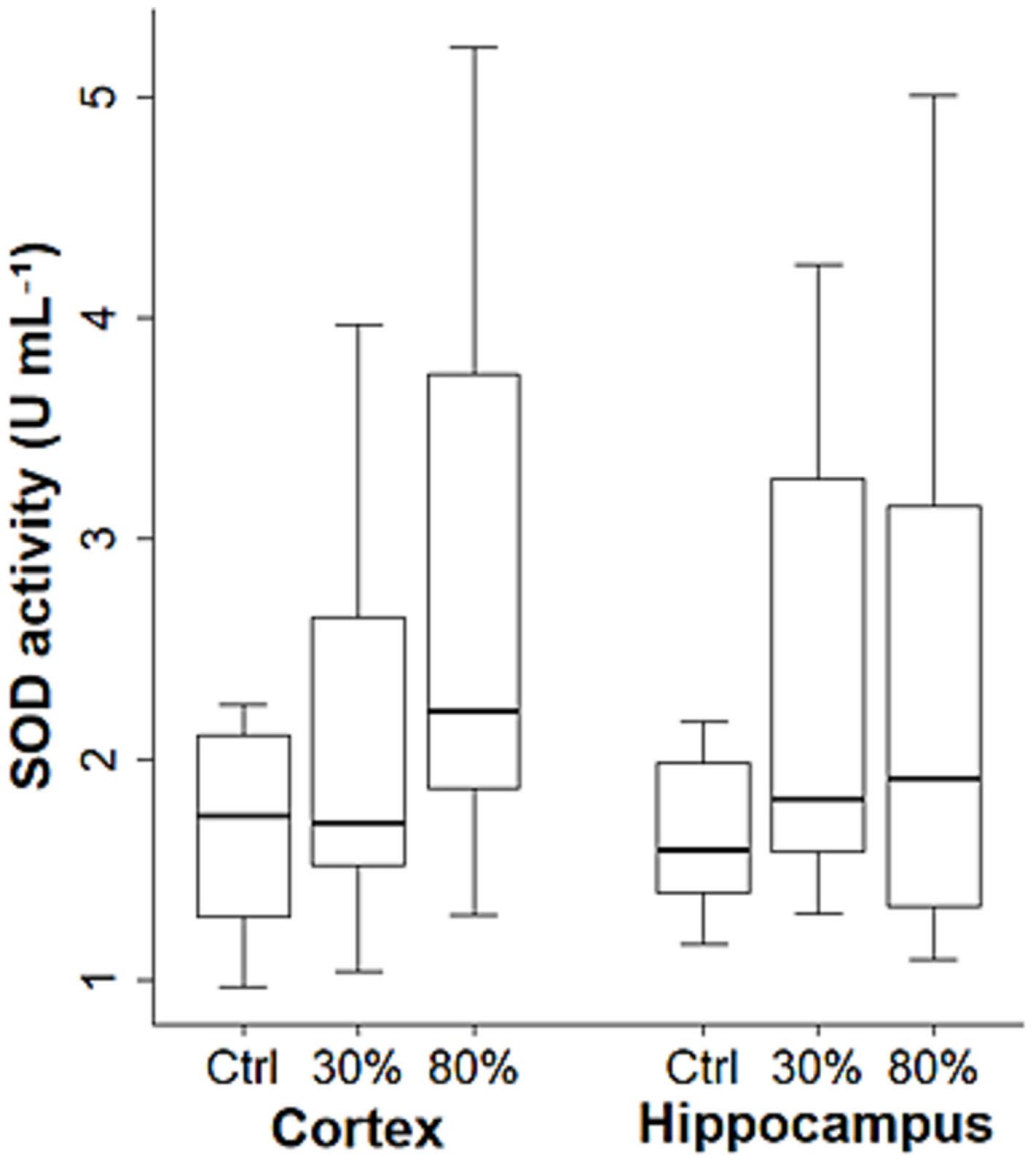
SOD activities based on oxygen fraction in all age groups (regardless of age). The data are presented as box plots. The horizontal box lines represent the 75th, 50th, and 25th percentiles. The stems represent the 90th and 10th percentiles. SOD, superoxide dismutase.

The cytokine levels and SOD activities based on the oxygen fraction in all age groups are shown in Figures 2, 3. Significant differences in IL-1β levels were observed in the hippocampus among the control, FiO₂ of 30%, and FiO₂ of 80% groups [2.3 (1.6–2.7) pg. mL^−1^ vs.3.2 (2.0–10.0) pg. mL^−1^ vs. 5.0 (4.0–6.9) pg. mL^−1^, respectively; *p* = 0.034]. A *post hoc* test revealed that IL-1β level in the hippocampus was significantly higher in the FiO₂ 80% group compared with controls [5.0 (4.0–6.9) pg. mL^−1^ vs. 2.3 (1.6–2.7) pg. mL^−1^, respectively; adjusted *p* = 0.032], whereas no significant differences were observed in IL-1β level between the other group pairs (control vs. FiO₂ 30%, adjusted *p* = 0.164; FiO₂ 30% vs. FiO₂ 80%, adjusted *p* = 0.390). Except IL-1β in the hippocampus, no significant differences in the cytokine levels and SOD activities were observed among the groups according to the inspired oxygen fraction (FiO₂ of 30%, FiO₂ of 80%, and control groups) in either the hippocampus or cortex (*p* > 0.050 for each).

Cytokine levels and SOD activity according to the oxygen fraction in young and old mice groups are presented in Tables 1, 2. No differences were observed in any of the cytokine levels or SOD activities in the cortex and hippocampus according to the oxygen fraction in the cortex and hippocampus in the young and old mice, respectively (*p* > 0.05 for each).

**Table 1:** Cytokine levels and SOD activities according oxygen fraction in young mice.

	Cortex				Hippocampus			
Variable	Control	FiO₂ 30%	FiO₂ 80%	*P*-value	Control	FiO₂ 30%	FiO₂ 80%	*P*-value
IFN-γ	22.8 (6.0–43.5)	20.1 (3.8–39.6)	22.6 (4.7–37.8)	0.849	37.9 (7.7–65.3)	23.2 (7.8–38.0)	32.8 (8.3–53.6)	0.557
IL-1α	115.1 (76.7–153.3)	100.4 (77.3–135.6)	103.0 (64.8–135.2)	0.676	136.8 (91.4–191.5)	117.7 (89.5–152.9)	125.9 (69.9–170.3)	0.444
IL-1β	2.3 (0.8–5.7)	2.8 (0.3–3.3)	14.9 (12.6–15.9)	0.085	2.5 (1.9–5.2)	2.7 (1.8–4.3)	13.6 (10.9–14.8)	0.183
IL-2	91.9 (30.2–152.7)	82.1 (28.1–142.8)	82.1 (25.0–140.2)	0.423	101.8 (32.4–172.7)	87.7 (31.3–168.9)	96.1 (28.3–162.5)	0.331
IL-4	1.4 (1.1–1.7)	1.1 (0.9–1.8)	1.6 (0.8–2.8)	0.747	1.8 (1.2–2.2)	1.4 (1.1–2.8)	2.2 (0.9–3.4)	0.92
IL-5	12.6 (11.1–12.8)	10.1 (8.1–11.1)	10.1 (9.5–10.7)	0.432	11.7 (10.9–12.4)	13.1 (10.3–13.9)	13.9 (10.8–13.9)	0.805
IL-6	27.1 (18.9–33.0)	26.0 (25.1–27.2)	28.2 (28.1–29.6)	0.797	37.8 (34.6–41.1)	27.7 (25.4–29.7)	31.9 (31.8–32.9)	0.141
IL-9	1638.3 (1119.1–2099.5)	1323.9 (1184.2–1767.5)	1647.6 (1120.6–2220.6)	0.927	1800.9 (1273.7–2482.7)	1661.6 (1499.2–2218.9)	2115.2 (1136.2–2970.5)	0.927
IL-12	6.4 (3.2–8.1)	8.1 (6.2–9.6)	9.6 (6.7–11.2)	0.757	2.4 (1.8–8.1)	8.1 (4.3–9.6)	9.7 (8.9–9.7)	0.558
IL-13	91.9 (35.3–126.3)	52.5 (25.0–116.5)	96.9 (33.0–147.9)	0.738	123.2 (37.4–213.3)	97.6 (36.9–201.5)	118.5 (51.8–179.6)	0.949
IL-17	2.5 (1.6–3.7)	0.8 (0.5–1.4)	2.3 (1.3–3.3)	0.285	4.2 (2.8–5.4)	4.6 (2.0–10.7)	4.6 (3.8–7.2)	0.996
TNF-α	4.4 (4.1–4.7)	3.7 (2.6–4.2)	5.2 (5.0–5.3)	0.057	4.5 (1.9–6.8)	4.7 (1.5–7.8)	6.7 (6.4–7.9)	0.584
SOD	1.3 (1.3–1.8)	1.6 (1.5–2.3)	2.0 (1.7–3.1)	0.202	1.3 (1.2–1.6)	1.6 (1.5–2.0)	1.3 (1.2–1.5)	0.271

IFN-γ, interferon-γ; IL, interleukin; TNF-α, tumor necrosis factor-α; SOD, superoxide dismutase.

Data are presented as median (interquartile range).

*p*-values were obtained using the Kruskal–Wallis test followed by Dunn’s post hoc pairwise comparisons, where appropriate.

All cytokine concentrations are expressed in pg mL^−1^, and SOD activity is expressed in U *mL^−1^*.

**Table 2:** Cytokine levels and SOD activities according oxygen fraction in old mice.

	Cortex				Hippocampus			
Variable	Control	FiO₂ 30%	FiO₂ 80%	*P*-value	Control	FiO₂ 30%	FiO₂ 80%	*P*-value
IFN-γ	6.0 (1.5–50.2)	7.8 (5.9–49.8)	3.7 (0.9–37.8)	0.329	30.8 (7.3–61.9)	12.4 (8.7–49.0)	9.4 (7.2–18.9)	0.583
IL-1α	80.7 (62.5–170.4)	91.9 (83.6–170.6)	82.8 (68.4–142.7)	0.519	92.6 (66.1–204.8)	106.0 (95.9–172.6)	87.6 (81.7–116.6)	0.219
IL-1β	3.0 (1.1–4.5)	5.1 (2.2–11.8)	2.2 (1.0–5.3)	0.326	1.8 (1.0–2.4)	3.4 (2.7–10.5)	4.4 (4.0–5.9)	0.061
IL-2	31.9 (13.9–161.6)	35.1 (27.1–157.8)	30.1 (25.6–148.8)	0.734	33.1 (15.1–171.7)	35.5 (34.6–150.2)	33.0 (32.2–66.7)	0.289
IL-4	1.0 (0.7–2.0)	0.9 (0.8–1.7)	0.9 (0.6–1.3)	0.937	1.0 (0.3–1.5)	1.1 (0.7–1.6)	0.6 (0.5–0.9)	0.240
IL-5	16.5 (15.6–17.7)	16.2 (13.2–17.2)	9.6 (8.6–10.4)	0.099	13.1 (6.2–13.9)	12.6 (9.4–15.7)	11.7 (10.9–11.9)	0.759
IL-6	11.3 (0.7–25.5)	14.8 (3.2–27.1)	15.5 (2.0–28.9)	0.52	30.4 (26.4–31.7)	5.5 (4.3–29.6)	3.5 (0.0–17.5)	0.119
IL-9	1212.8 (582.1–2444.6)	2116.2 (1327.8–2486.2)	2360.1 (1195.8–2588.2)	0.301	1606.9 (487.4–2605.7)	2532.2 (1513.3–3320.3)	2821.7 (809.3–3498.2)	0.127
IL-12	18.6 (17.4–18.6)	11.2 (2.8–13.8)	8.1 (0.9–8.1)	0.132	8.1 (6.7–8.9)	4.3 (1.1–11.2)	9.6 (8.9–10.4)	0.743
IL-13	55.7 (31.6–179.8)	136.8 (38.7–166.6)	141.1 (3.9–152.2)	0.946	51.1 (32.3–251.0)	211.8 (52.2–255.1)	181.8 (109.0–219.8)	0.609
IL-17	2.8 (2.6–3.3)	2.0 (1.3–2.8)	2.3 (1.5–3.3)	0.917	4.0 (1.6–4.7)	3.2 (2.3–4.0)	1.9 (1.3–3.0)	0.193
TNF-α	5.5 (4.8–5.8)	4.8 (0.9–5.4)	5.5 (5.3–6.4)	0.418	5.4 (2.7–8.0)	6.6 (2.0–6.9)	7.7 (0.0–7.8)	0.858
SOD	1.8 (1.6–4.2)	1.9 (0.7–4.0)	2.9 (2.1–4.3)	0.429	1.8 (1.6–4.0)	2.2 (1.6–4.1)	2.5 (0.9–3.6)	0.653

IFN-γ, interferon-γ; IL, interleukin; TNF-α, tumor necrosis factor-α; SOD, superoxide dismutase.

Data are presented as median (interquartile range).

*P*-values were obtained using the Kruskal–Wallis test followed by Dunn’s post hoc pairwise comparisons, where appropriate.

All cytokine concentrations are expressed in pg mL^−1^, and SOD activity is expressed in U mL^−1^.

Additionally, no significant differences in cytokine levels and SOD activity were observed between the young and old mice at each inspired oxygen concentration (*p* > 0.05 for each).

## Discussion

This study demonstrated that the FiO₂ 80% group during anesthesia presented significantly higher IL-1β levels in the hippocampus compared with the control group (FiO₂ of 21%). However, IL-1β levels did not differ between the control and FiO₂ 30% groups and between the FiO₂ 30% and FiO₂ 80% groups. Except for IL-1β in the hippocampus, other cytokine levels other than IL-1β and SOD activities in the cortex and hippocampus did not differ among the control, FiO₂ 30%, and FiO₂ 80% groups during anesthesia.

Although a broad panel of cytokines and antioxidant markers was assessed, IL-1β in the hippocampus was the only marker showing a statistically significant group difference, and thus it is discussed as the primary positive finding of the study. Based on our finding that IL-1β levels in the hippocampus were significantly higher in the 80% oxygen group compared with normoxia, exposure to 80% supraphysiological oxygen may be associated with a modest and selective increase in IL-1β levels in the hippocampus, rather than a global neuroinflammatory response. Oxidative stress is closely linked to the upregulation of pro-inflammatory mediators in the brain, and IL-1β is known to be a pro-inflammatory cytokine that has been implicated in neuronal injury, synaptic dysfunction, and cognitive impairment (Buffolo et al., 2021; Li Puma et al., 2023; Fogal et al., 2005). However, no significant difference in IL-1β level between the 30 and 80% oxygen groups suggests that an FiO₂ of 30% or 80%, conventionally used in the clinical anesthesia, may not lead to robust or consistently detectable differences in neuroinflammatory or antioxidant markers under the present experimental conditions. Importantly, subtle inflammatory or redox changes may not have been detectable under the present experimental conditions, and limited statistical power may have contributed to the discrepancy between the significant finding in hippocampal IL-1β and the largely negative findings across other markers.

Our findings that except for IL-1β in the hippocampus, no significant differences were observed in the cytokines and antioxidant activity in the cortex and hippocampus among the control, FiO₂ 30%, and FiO₂ 80% groups were inconsistent with previous studies investigating the effect of hyperoxia on the brain (Hencz et al., 2024; Onodera et al., 2003). An experimental study showed that exposure to 100% oxygen for 48 h significantly increased antioxidant enzyme activities including SOD and glutathione peroxidase in the cerebral cortex and hippocampus compared to the control group (exposure to 20% oxygen) (Onodera et al., 2003). Another study assessing the effects of 1 h exposure to mild hyperoxia (30%) or severe hyperoxia (100%) on rat hippocampal neurons also suggested that both mild and severe hyperoxia increased functional and morphological changes in the hippocampus (Hencz et al., 2024). However, hyperoxemia does not necessarily translate into tissue hyperoxia because of the low solubility of oxygen in the blood, limited buffering capacity of hemoglobin, and cerebral autoregulation, which together prevent excessive oxygen delivery to the brain tissue (Diop et al., 2025; Bucci, 2009; Jackson, 2016; Lång et al., 2018). Prior studies that directly measured brain tissue oxygen tension have shown that increases in inspired oxygen can elevate cerebral oxygen tension in injured brain, although the magnitude and uniformity of this effect vary with physiological conditions and tissue state (). Hyperoxia increased brain tissue oxygen tension in patients with focal lesions (Longhi et al., 2002) and in pediatric traumatic brain injury (Figaji et al., 2010), but the response was strongly influenced by regional blood flow and baseline tissue perfusion (Hlatky et al., 2008). These findings indicate that meaningful cerebral hyperoxia may occur under some conditions, but the extent and relevance in short-term anesthesia without pathology remain uncertain. Furthermore, previous studies have shown that hyperoxia aggravates oxidative stress and neuroinflammation in vulnerable conditions, such as traumatic brain injury, immature brain, and global ischemia–reperfusion injury (Felderhoff-Mueser et al., 2004; Blasiole et al., 2013; Richards et al., 2007; Hazelton et al., 2010). In the present study, pathological or traumatic experimental conditions were not applied, although aged mice were included. Therefore, inflammation or SOD activity in the cortex or hippocampus may be less affected by hyperoxemia. Several perioperative clinical trials have consistently shown that varying inspired oxygen concentrations during surgery does not result in differences in postoperative cognitive outcomes. A randomized trial involving elderly patients undergoing cardiac surgery showed no significant differences in postoperative cognitive outcomes between the 35 and 100% FiO₂ groups (Shaefi et al., 2021). Similarly, in a study involving elderly patients undergoing laparoscopic gastric or colorectal cancer surgery, the incidence of postoperative delirium was comparable between the 40 and 80% FiO₂ groups (Lin et al., 2021). In another randomized trial including elderly patients undergoing major abdominal surgery, there was no significant difference in delirium between the 40 and 100% FiO₂ groups, and postoperative plasma neuroinflammatory markers (IL-6, TNF-*α*, neuron-specific enolase, and neurofilament light chain) were also comparable between the different inspired oxygen groups (Ji et al., 2024).

In the present study, both young and old mice showed no significant difference in cytokine levels or SOD activity among the different inspired oxygen concentrations applied during anesthesia, although previous studies have shown that aging enhances baseline oxidative stress and alters immune responses (Mariani et al., 2005; Floyd and Hensley, 2002). Our experimental conditions may not have been sufficiently sensitive to detect subtle age-related changes in neuroinflammatory or redox markers. Notably, age-related alterations in redox homeostasis may occur even in the absence of overt neuroinflammation, as demonstrated by prior work showing changes in hippocampal antioxidant enzyme activity with aging (Lacoste et al., 2017). Experimental studies with clearly defined age groups have suggested that neuroinflammatory changes, reflected by enhanced glial and microglial activation, become more pronounced at later stages of the murine lifespan. Specifically, compared with young adult mice (5–6 months), aged mice (21–24 months) exhibited exaggerated glial responses following injury in the hippocampus (Sandhir et al., 2008). Similarly, 24-month-old mice showed higher hippocampal expression of microglial activation markers than 6-and 12-month-old mice (Shin et al., 2025). In contrast, the 18-month time point examined in the present study likely represents an earlier stage of aging, at which biological alterations may be modest and potentially below the detection threshold of the assays employed. Accordingly, the largely negative age-dependent findings observed here should be interpreted in the context of aging stage and methodological sensitivity. Thus, the lack of clear age-dependent differences in the present study does not preclude the presence of more subtle age-related redox alterations under different or more challenging experimental conditions.

Mice aged 18–24 months of age are generally considered old, as mice between the ages of 18 and 24 months correlated with humans ranging from 56 to 69 years of age (Flurkey et al., 2007). The mice used in this study were 18 months old, which corresponds to a human age of 56 years. Thus, the ‘old’ mice in the present study may represent an early stage of aging rather than advanced senescence. Therefore, further studies on the effects of hyperoxia during anesthesia on neuroinflammation and oxidative stress in older mice are required.

This study had several limitations. First, the brain tissue oxygenation was not directly measured. *Such measurements would clearly determine* the relationship between high-inspired oxygen exposure and the occurrence of brain hyperoxia and oxidative stress. Second, our study evaluated 3 h of oxygen exposure during general anesthesia without surgical intervention. As surgical stress and longer exposure durations may influence physiological responses, the generalizability of our findings to surgical settings may be limited. Third, in addition to the molecular markers assessed in the brain tissue, histopathological analyses and behavioral testing would have provided more concrete evidence regarding the impact of oxygen exposure on structural brain changes and neurocognitive outcomes. Fourth, the relatively small sample size may have contributed to the apparent variability in the data and reduced the statistical power for detecting group differences. Finally, the ‘old’ mice used in this study (18 months) may not fully represent advanced aging. More advanced-aged animals (e.g., ≥24 months) may exhibit greater vulnerability to hyperoxia-induced neuroinflammatory or redox alterations, and future studies using older mice are warranted.

In conclusion, 80% oxygen exposure during anesthesia induced a significant increase in IL-1β levels in the hippocampus compared with medical air (FiO₂ 21%), suggesting region-specific vulnerability of the hippocampus to oxygen-induced neuroinflammation. However, considering that there was no difference in IL-1β levels between the FiO₂ 80% and FiO₂ 30% groups and between the FiO₂ 30% and control groups, it may have no significant inflammatory effect in the hippocampus in terms of clinical oxygen management during anesthesia. In addition, no significant differences were observed in other cytokines and SOD activity in the cortex and hippocampus among the control, FiO₂ 30%, and FiO₂ 80% groups in the young and old mice. Responses to different inspired oxygen levels during anesthesia did not differ between the young and old mice. Further studies with larger sample sizes and more comprehensive analyses are needed to clarify the impact of high oxygen exposure on the brain during anesthesia and surgery.

## Data Availability

The raw data supporting the conclusions of this article will be made available by the authors, without undue reservation.
